# Automated extraction of information of lung cancer staging from unstructured reports of PET-CT interpretation: natural language processing with deep-learning

**DOI:** 10.1186/s12911-022-01975-7

**Published:** 2022-09-01

**Authors:** Hyung Jun Park, Namu Park, Jang Ho Lee, Myeong Geun Choi, Jin-Sook Ryu, Min Song, Chang-Min Choi

**Affiliations:** 1 Department of Pulmonary and Critical Care Medicine, Asan Medical Center, University of Ulsan College of Medicine, 88, Olympic-ro 43-gil, Songpa-gu, Seoul, 05505 South Korea; 2 Department of Biomedical Informatics and Medical Education, School of Medicine, University of Washington, Seattle, WA USA; 3 Division of Pulmonary and Critical Care Medicine, Department of Internal Medicine, College of Medicine, Mokdong Hospital, Ewha Womans University, Seoul, Republic of Korea; 4 Department of Nuclear Medicine, Asan Medical Center, University of Ulsan College of Medicine, Seoul, Korea; 5 Department of Digital Analytics, Yonsei University, 50 Yonsei-ro, Seodaemun-gu, Seoul, 03722 South Korea; 6 Department of Oncology, Asan Medical Center, University of Ulsan College of Medicine, Seoul, South Korea; 7 Department of Information Medicine, Asan Medical Center, University of Ulsan College of Medicine, Seoul, South Korea

**Keywords:** Natural language processing, Auto-annotation, Deep learning, Lung cancer, Pseudo-labelling

## Abstract

**Background:**

Extracting metastatic information from previous radiologic-text reports is important, however, laborious annotations have limited the usability of these texts. We developed a deep-learning model for extracting primary lung cancer sites and metastatic lymph nodes and distant metastasis information from PET-CT reports for determining lung cancer stages.

**Methods:**

PET-CT reports, fully written in English, were acquired from two cohorts of patients with lung cancer who were diagnosed at a tertiary hospital between January 2004 and March 2020. One cohort of 20,466 PET-CT reports was used for training and the validation set, and the other cohort of 4190 PET-CT reports was used for an additional-test set. A pre-processing model (Lung Cancer Spell Checker) was applied to correct the typographical errors, and pseudo-labelling was used for training the model. The deep-learning model was constructed using the Convolutional-Recurrent Neural Network. The performance metrics for the prediction model were accuracy, precision, sensitivity, micro-AUROC, and AUPRC.

**Results:**

For the extraction of primary lung cancer location, the model showed a micro-AUROC of 0.913 and 0.946 in the validation set and the additional-test set, respectively. For metastatic lymph nodes, the model showed a sensitivity of 0.827 and a specificity of 0.960. In predicting distant metastasis, the model showed a micro-AUROC of 0.944 and 0.950 in the validation and the additional-test set, respectively.

**Conclusion:**

Our deep-learning method could be used for extracting lung cancer stage information from PET-CT reports and may facilitate lung cancer studies by alleviating laborious annotation by clinicians.

**Supplementary Information:**

The online version contains supplementary material available at 10.1186/s12911-022-01975-7.

## Introduction

Medical big-data analysis could use deep learning to reveal novel associations between treatment and patient factors, and potential risk groups [1, 2]. However, most electronic health data are currently stored in unstructured language forms such as clinical reports and radiologic reports, which require manual review by clinicians or radiologists in order to be transformed into structured datasets ready for analysis [1]. Therefore, automation of the review and annotation of unstructured health reports would be helpful. With the development and application of the deep-learning method in text mining [2] and natural language processing [3], there have been several attempts for the automatic classification of medical records such as the extraction of diagnoses from radiologic reports [4, 5], automatic coding of ICD-9 or 10 from medical chart [6–8], and extraction of tumour type, size, and location from colonoscopic reports [9]. In terms of positron emission tomography-computed tomography (PET-CT) reports, previous studies have sought to determine the presence of lymphoma involving bone [10] and the treatment response of lymphoma [11]. Despite some success from automatic extraction, clinical studies continue to rely on manual chart review as they require more specific information.

When conducting studies on lung cancer, clinicians mostly obtain information regarding the primary sites from chest CT reports [12], and information on cancer staging from PET-CT with 18F-fluorodeoxyglucose. [13] The extraction of distant metastases and the staging of the lung cancer itself are vital when choosing the appropriate oncological therapy and predicting patient prognosis. [14] Also, identifying metastatic sites such as the liver [13] or spine [15] provides valuable prognostic information as well. Even though the amount of information on PET-CT is increasing due to its increasing usage, only a small proportion of such information could be automatically extracted due to the unstructured nature of text data.

Auto-extraction from reports written by natural language is essential. However, annotated datasets are needed in order to build an auto-extraction model. In contrast to classifying radiologic images, annotating all metastatic sites from text-based reports is a highly laborious process that may entail inaccurate annotation. Moreover, raw data of free-texted reports have many typographical errors and different writing styles across radiologists, which lowers the accuracy of deep-learning models.

In this study, we sought to overcome these barriers by developing a spelling correction tool for the lung cancer domain that served as a pre-processing tool for radiology reports and implemented a semi-supervised learning method called pseudo-labelling during the training process [16]. With this technique, we devised a deep-learning model for extracting the primary location of lung cancer sites and metastatic lymph nodes and distant metastatic sites from PET-CT reports consisting of unstructured natural language.

## Methods

### Clinical data

We collected the PET-CT reports of patients who were diagnosed with lung cancer between January 1st, 2007, and March 31st, 2020 at Asan Medical Center, a tertiary referral hospital in Seoul, South Korea (Cohort A). The records collected from patients with lung cancer were coded by the International Classification of Disease, 10th revision. The PET-CT reports consisted of the following data: patient ID, exam code, exam date, clinical diagnosis, the reason for an imaging study, examination methods, description of image findings, and conclusion of image interpretation. The conclusion section of the report, written in English, would contain the locations of the primary cancer site, the metastatic lymph nodes, and other metastatic lesions. Additional file 1: Figure S1 shows an example of the conclusion section of a PET-CT report from a patient with lung cancer that was used as the input data in this study. To evaluate the performance of the generated model in the additional-test set, we used PET-CT reports of patients from a different cohort at Asan Medical Center who were treated between January 1st, 2004, and March 31st, 2020 (Cohort B). Although the additional-test set was not collected from different hospital records, we intended to show that our model can work on independent annotated datasets without any overlap in patients. The purpose of our model was to convert any lung cancer PET-CT reports into a structured form so that clinicians could access the metastasis-labelled radiologic reports.

### Report annotation

To determine the metastatic stage of lung cancer according to the TNM stage [17], we assessed the primary cancer location, nodal stage of lung cancer, and metastatic sites as the outcome categories. The location of lung cancer was labelled in the class of the lobe; however, if the primary site could not be determined by each lobe due to the huge size, the location was labelled as left or right. In the case of synchronous metastasis and ipsilateral/contralateral metastasis, the annotator follows the initial opinion of radiologists who reported the PET-CT reports. Two clinicians independently annotated the primary cancer location and the metastatic lymph nodes and organs in 500 PET-CT reports and their consistency was calculated by Cohen’s kappa coefficient (Additional file 1: Table S1). Another clinician independently annotated the primary cancer location and metastatic organs in 4190 PET-CT reports that were used as the additional-test set. The additional-test dataset was not used in the pseudo-labelling process nor in any pre-processing.

### Ethics approval

The ethics committee of Asan Medical Center approved this study, conducted following the declaration of Helsinki. Also, the ethics committee of Asan Medical Center (approval number 2020–0212) waived the informed consent due to the retrospective observational nature of the study. The clinical data extracted using the ABLE system at Asan Medical Center were indexed by de-identified encrypted patient ID numbers so that the individual patients could not be identified [18, 19].

### Pre-processing of typographical errors and keyword extraction

In order to train a deep-learning model that is robust against typographical errors, we developed a spelling correction tool trained on lung cancer-related journals (Additional file 1: Methods). All the radiologic reports had been corrected using this spelling correction tool. As each sentence had an independent meaning in our PET-CT reports, each radiologic report was split into a group of sentences (Additional file 1: Figure S1). Keywords were extracted from each sentence using Named Entity Recognition (NER) [20], which eliminates words that had less impact on extracting the metastatic information. (Additional file 1: Figure S2) Eventually, the whole pre-processing stage provides a refined version of the input data that have been transformed into a set of sentences containing keywords; in turn, the pre-processed inputs are used to train the deep-learning models. The detailed methods for pre-processing were described in Additional file 1: Methods.

### Structure of the model

**Fig. 1:**
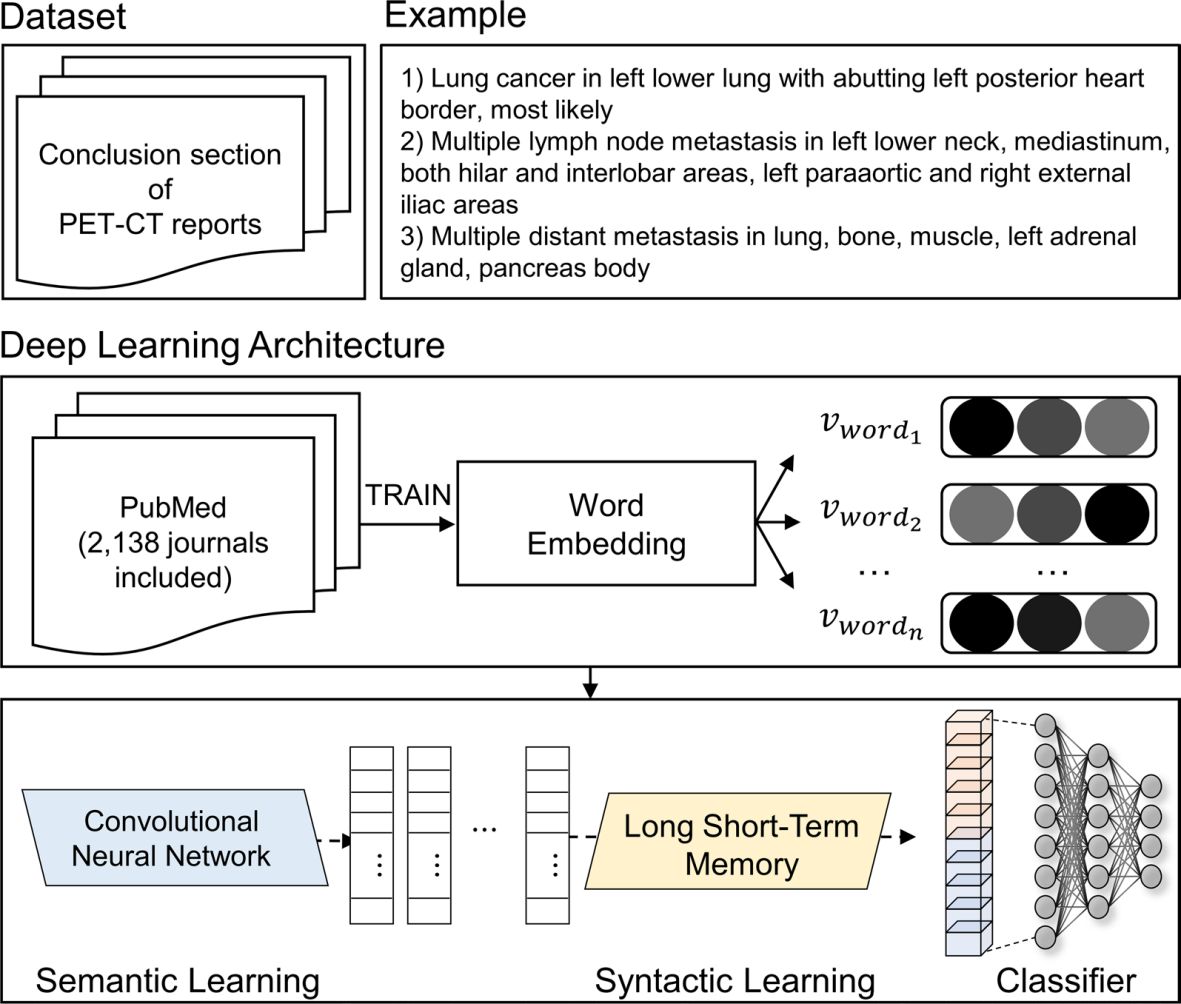
Structure of the model pre-processing and development for extracting information

Using the NER tags, we extracted keywords that might represent the primary sites from each PET-CT report. Each keyword consisted of 100-dimensional vectors. In this study, we implemented the Convolutional-Recurrent Neural Network [21] consisting of a single convolutional layer and two LSTM layers (Fig. 1). Convolutional operation and max-pooling extracts key features within the FastText embedding, while LSTM operation focuses on sequential information among the word sequence. This method could improve the representation of words that reflect their context as well as the semantics.

The classification of primary sites is a multi-class classification task, while lymph node staging is a multi-label classification task. The primary cancer sites are listed as the right upper lobe, right middle lobe, right lower lobe, left upper lobe and left lower lobe—a multi-class classification. The lymph node stage is determined by the most distant metastatic lymph nodes from the primary cancer location (TNM staging). Therefore, the model should find all the metastatic lymph nodes, identify the anatomical site and determine whether it is ipsilateral or contralateral with respect to the primary cancer site. In annotating these metastatic lymph nodes, there are some problems. First, too many labels should be annotated for one report, which could lead to the omission of some label annotation by clinicians. The second is the long-tailed distribution of metastatic sites, such that only a small number of uncommon metastatic sites are extracted despite the laborious process of annotation. To overcome this hurdle, target sites for lymph nodes and metastatic organs were selected if their prevalence was higher than 3%. We also used a semi-supervised learning technique called pseudo-labelling, which first trains the model using the small number of labelled data and then assigns pseudo-labels that shows the highest probability to the unlabelled data using that model. Although this method is a relatively simple approach, it showed high performance compared with other semi-supervised learning methods [16]. Using this approach, we assigned pseudo-labels to every unlabelled data; however, unlike in the original paper on pseudo-labelling [16], each pseudo-label was assigned considering the appearance of specific words, not based on probabilistic values. For instance, sentences in which ‘hilar’ and ‘metastasis’ appear are most often related to metastasis in the hilar area, so its pseudo-label would be ‘metastatic hilar lymph nodes.’ As the label value was closely related to the extracted information, keywords within each sentence were used to return pseudo-labels for multi-label classification.

The nodal (N) staging classification model has 13 outputs corresponding to the number of categories belonging to the N stage, and the metastatic sites (M) stage classification model has seven final nodes corresponding to each category. As lymph node staging is determined by the most distant lymph nodes, and by whether the lymph node is ipsilateral or contralateral to primary sites, further processing was necessary in order to determine the location of the metastatic lymph nodes and the primary cancer site. Using the keywords that were used during the pseudo-labelling process, we checked the n-grams surrounding each keyword with the purpose of considering the closest positional word (ipsilateral or contralateral). Next, we analysed the word segments containing the keywords as well as the location information, with the primary cancer site in order to determine the side of the lymph node. (Additional file 1: Figure S3) In nodal and distant metastasis staging, the extraction model uses all words, not just keywords, which would help the model learn other expressions that are not included in the keywords. Accordingly, we noticed that the model was appropriately trained as the sentences containing words such as ‘T4’ and ‘T5’—abbreviation of ‘4th and 5th thoracic vertebrae’—tend not to contain words related to the bone. Therefore, all words were used as input in our proposed model.

### Statistical analysis

The prevalence of the outcome was described in numbers and percentages. The inter-rater agreement was calculated by Cohen’s kappa coefficient and the overall accuracy of our proposed model with each pre-processing was described with the A/B test. The performance of our proposed model was evaluated with the following metrics: precision, sensitivity (recall), specificity, F_1_- score, area under the receiver operating curve (AUROC), and area under the precision-recall curve (AUPRC) with micro-average and macro-average for each outcome in the two validation sets. [22] For each outcome, we evaluated the false-positive and false negative results according to each label. Statistical analysis was performed by the statistics package in Python 3.7.4.

## Results

A total of 20,466 PET-CT reports were collected in Cohort A, of which 19,466 inputs were used in our model for extracting keywords and pseudo-label training. After excluding 27 reports that had more than two primary locations of cancer, 473 reports annotated by clinicians were used as the validation set for evaluating the primary sites and lymph node and metastatic organs. For additional-test in Cohort B, 3362 PET-CT reports were used for validating the primary sites and metastatic organs after excluding 828 reports in which there were more than two primary lung cancers, or the primary cancer was not lung cancer (Table 1). The number of metastatic lymph nodes and organs had a prevalence ranging from 0.1% (scalene lymph node) to 25% (bone).

**Table 1:** Prevalence of primary sites and metastatic lymph nodes and organs

	Validation set (N = 473)		Additional-test set (N = 3362)	
	Number	Prevalence	Number	Prevalence
*Primary lung lesion area*				
Left				
Left (huge)	6/473	0.0127	143/3362	0.0425
Left lower lobe	84/473	0.1776	553/3362	0.1645
Left upper lobe	126/473	0.2664	765/3362	0.2275
Right				
Right (huge)	8/473	0.0169	171/3362	0.0509
Right lower lobe	90/473	0.1903	690/3362	0.2052
Right middle lobe	27/473	0.0571	191/3362	0.0568
Right upper lobe	132/473	0.2791	849/3362	0.2525
*Lymph node*				
N1				
Hilar	142/473	0.3002		
Interlobar	123/473	0.2600		
(Peri) Bronchial	3/473	0.0063		
Lobar	6/473	0.0127		
N2				
Upper paratracheal	32/473	0.0677		
Prevascular, retrotracheal	36/473	0.0761		
Lower paratracheal	77/473	0.1628		
Subaortic	10/473	0.0211		
Para-aortic	23/473	0.0486		
Subcarinal	65/473	0.1374		
Para-oesophageal	19/473	0.0402		
N3				
Contralateral N1	43/473	0.0909		
Contralateral N2	87/473	0.1839		
Supraclavicular	99/473	0.2093		
*Metastasis*				
Intra-thoracic metastasis				
Malignant pleural effusion	36/473	0.0761	342/3362	0.1017
Malignant pericardial effusion	6/473	0.0127	20/3362	0.0059
Pleural nodule	55/473	0.1163	542/3362	0.1612
Contralateral lung	61/473	0.1290	363/3362	0.1080
Ipsilateral lung	65/473	0.1374	1/3362	0.0003
Synchronous lung cancer	11/473	0.0233	13/3362	0.0039
Lymphangitic meta	9/473	0.0190	46/3362	0.0137
Extra-thoracic metastasis				
Bone (including rib and sternum)	119/473	0.2516	697/3362	0.2073
Extra-thoracic lymph node	82/473	0.1734	401/3362	0.1193
Brain	14/473	0.0296	75/3362	0.0223
Adrenal	22/473	0.0465	177/3362	0.0526
Liver	42/473	0.0888	62/3362	0.0184
Other	46/473	0.0973	143/3362	0.0425

### Evaluation of primary cancer location classification

**Fig. 2:**
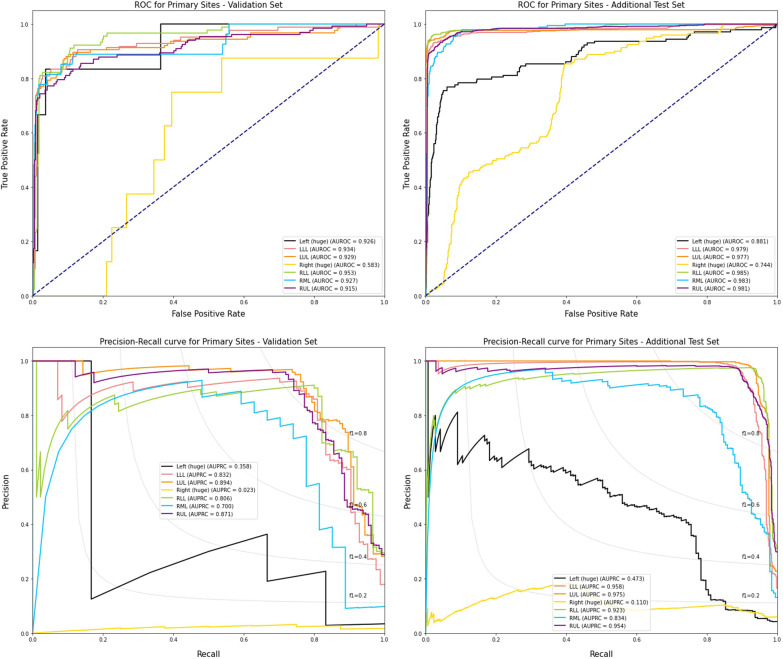
AUROC and AUPRC curves for the prediction of primary sites in the validation set and the additional-test set. The AUROC and AUPRC curves on the validation set and the additional-test set are shown. The AUROC values for primary cancer sites were 0.913 and 0.946 in the validation set and the additional-test set, respectively. The micro-AUPRC values for primary cancer sites were 0.819 and 0.902 in the validation set and the additional-test set, respectively. F_1_: line of F_1_-score with some value (0.2 to 0.8)

In primary site classification among the 473 reports, the overall precision and sensitivity were 0.795 and 0.774, respectively, and micro-AUROC and weighted-AUROC were 0.913 and 0.924, respectively. The precision/sensitivity and AUROC and AUPRC per site are shown in Table 2 and Fig. 2. In the 3362 additional-test sets that had only one primary lung cancer, the overall precision and sensitivity were 0.831 and 0.850, respectively, and micro-AUROC and weighted-AUROC were 0.946 and 0.965, respectively. Low performance was observed in the prediction of a huge-sized left or right lobe in which the lobar location could not be defined due to invading the boundary of the lobe. In the validation set, when the model target was only considered between the left and right lobe, which is important information for deciding ipsilateral lymph nodes, the precision and sensitivity for the left lobe were 0.894 and 0.898, respectively, while those for the right lobe were higher at 0.914 and 0.911, respectively. In the additional-test set, the precision and sensitivity were 0.967 and 0.949 for the left lobe, respectively, and 0.962 and 0.975 for the right lobe, respectively.

**Table 2:** Prediction accuracy for primary cancer location and metastatic sites

	Validation set				Additional-test set			
	Frequency	Precision	Sensitivity	F_1_-Score	Frequency	Precision	Sensitivity	F_1_-Score
*Primary lung lesion*								
Left								
Left (huge)	6	0.2000 (1/5)	0.1667 (1/6)	0.1818	143	0.6786 (19/28)	0.1329 (19/143)	0.2222
Left lower lobe	84	0.7143 (70/98)	0.8333 (70/84)	0.7692	553	0.7894 (521/660)	0.9421 (521/553)	0.8590
Left upper lobe	126	0.8684 (99/114)	0.7857 (99/126)	0.8250	765	0.9598 (717/747)	0.9373 (717/765)	0.9484
Any of left†	216	0.8940 (194/217)	0.8981 (194/216)	0.8961	1461	0.9666 (1387/1435)	0.9493 (1387/1461)	0.9579
Right								
Right (huge)	8	0.0000 (0/0)	0.0000 (0/8)	0.0000	171	0.0000 (0/0)	0.0000 (0/171)	0.0000
Right lower lobe	90	0.8690 (73/84)	0.8111 (73/90)	0.8391	690	0.9361 (659/704)	0.9551 (659/690)	0.9455
Right middle lobe	27	0.3898 (23/59)	0.8519 (23/27)	0.5349	191	0.4360 (177/406)	0.9267 (177/191)	0.5930
Right upper lobe	132	0.8850 (100/113)	0.7576 (100/132)	0.8163	849	0.9376 (766/817)	0.9022 (766/849)	0.9196
Any of right†	257	0.9141 (234/256)	0.9105 (234/257)	0.9123	1901	0.9616 (1853/1927)	0.9748 (1853/1901)	0.9681
Overall	473	0.7953	0.7738	0.7767	3362	0.8308	0.8504	0.8265
*Metastatic organ*								
Intra-thoracic								
Malignant effusion	36	0.4096 (34/83)	0.9444 (34/36)	0.5714	342	0.57 (334/586)	0.9766 (334/342)	0.7198
Pleural nodule	55	0.6296 (51/81)	0.9273 (51/55)	0.7500	542	0.7674 (508/662)	0.9373 (508/542)	0.8439
Contralateral metastasis	61	0.3846 (40/104)	0.6557 (40/61)	0.4848	363	0.3441 (287/834)	0.7906 (287/363)	0.4795
Extra-thoracic								
Bone	119	0.8298 (117/141)	0.9832 (117/119)	0.9000	697	0.7462 (682/914)	0.9785 (682/697)	0.8467
Extra-thoracic LN‡	82	0.4530 (82/181)	1.0000 (82/82)	0.6236	401	0.3347 (399/1192)	0.995 (399/401)	0.5009
Adrenal	22	0.4872 (19/39)	0.8636 (19/22)	0.6230	177	0.4958 (175/353)	0.9887 (175/177)	0.6604
Liver	42	0.8810 (37/42)	0.8810 (37/42)	0.8810	62	0.2609 (12/46)	0.1935 (12/62)	0.2222
Overall	473	0.6150	0.9113	0.7202	3362	0.5782	0.9276	0.6963

^†^Predicting cancer site between right or left that do not consider subdivision of the lung lobes. ‡LN: lymph nodes

### Evaluation of node and distant metastasis

Using the validation data containing 473 radiology reports, we first evaluated the accuracy of our nodal and distant metastasis staging. As noted in the methods section, we started this process by checking the location of lymph nodes and then considered whether the side was ipsilateral or contralateral to the primary site. In the first phase, the overall precision and sensitivity for metastatic lymph nodes were 0.766 and 0.827, respectively, and the performance metrics for each lymph node are described in Table 3. Except for contralateral N1 and N2, lymph nodes with higher incidence had higher precision and sensitivity than those with lower incidence.

**Table 3:** Accuracy for prediction of metastatic lymph nodes in the validation set

Lymph node	Frequency	Precision	Sensitivity	Specificity	F_1_-score
*N1*					
Hilar	141	0.8141 (127/156)	0.9007 (127/141)	0.9127 (303/332)	0.8552
Interlobar	121	0.7740 (113/146)	0.9339 (113/121)	0.9062 (319/352)	0.8464
Lobar	6	0.4286 (6/14)	1.0000 (6/6)	0.9829 (459/467)	0.6000
*N2*					
Upper paratracheal	32	0.8696 (20/23)	0.6250 (20/32)	0.9932 (438/441)	0.7273
Prevascular, retrotracheal	35	0.9259 (25/27)	0.7143 (25/35)	0.9954 (436/438)	0.8065
Lower paratracheal	77	0.8533 (64/75)	0.8312 (64/77)	0.9722 (385/396)	0.8421
Subaortic	10	0.6000 (9/15)	0.9000 (9/10)	0.9870 (457/463)	0.7200
Para-aortic	23	0.6000 (21/35)	0.9130 (21/23)	0.9689 (436/450)	0.7241
Subcarinal	65	0.8000 (60/75)	0.9231 (60/65)	0.9632 (393/408)	0.8571
Para-oesophageal	19	0.7083 (17/24)	0.8947 (17/19)	0.9846 (447/454)	0.7907
*N3*					
Contralateral N1	61	0.5357 (45/84)	0.7377 (45/61)	0.9053 (373/412)	0.6207
Contralateral N2	109	0.7647 (52/68)	0.4771 (52/109)	0.9560 (348/364)	0.5876
Supraclavicular	99	0.9307 (94/101)	0.9495 (94/99)	0.981 (367/374)	0.9400
*Overall*		0.7663	0.8265	0.9603	0.7862

**Fig. 3:**
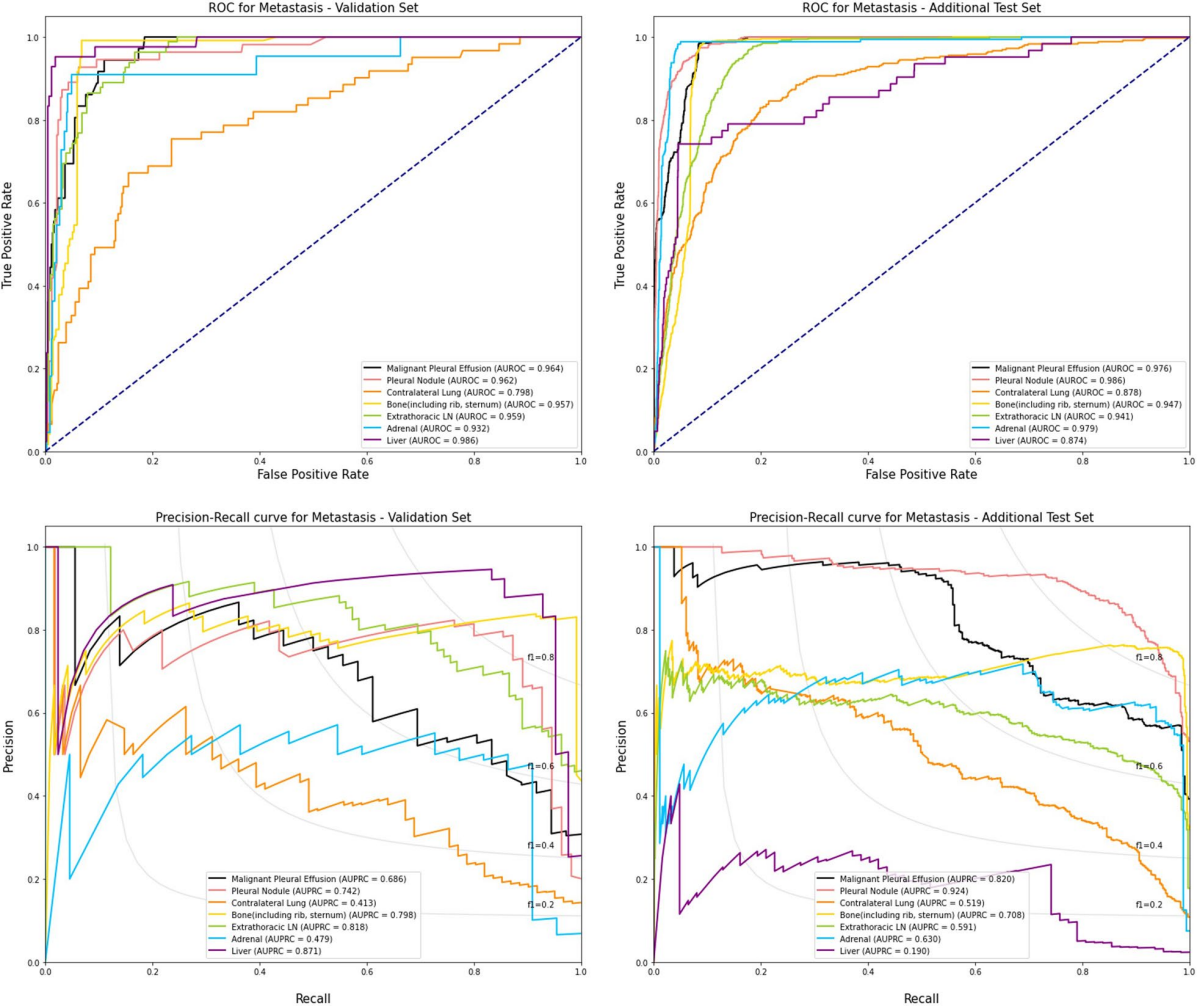
AUROC and AUPRC curves for predicting metastatic organs in the validation set and the additional-test set. The AUROC and AUPRC curves on the validation set and the additional-test set are shown. The AUROC values for predicting metastatic organs were 0.944 and 0.950 in the validation set and the additional-test set, respectively. The AUPRC values for metastatic organs were 0.687 and 0.640 in the validation set and the additional-test set, respectively. F_1_: line of F_1_-score with some value (0.2 to 0.8)

In terms of distant metastasis, the overall precision and sensitivity of the model were 0.615 and 0.911 in the validation set, respectively, and 0.578 and 0.928 in the additional-test set, respectively. In terms of the AUROC of metastatic organ prediction, micro-AUROC and weighted-AUROC were 0.944 and 0.937 in the validation set, respectively, and 0.950 and 0.949 in the additional-test set, respectively (Fig. 3). For each metastatic organ, the performance was the lowest in predicting contralateral lung metastasis in the validation set (F_1_-score = 0.489) and liver metastasis in the additional-test set (F_1_-score = 0.222), and the highest in predicting bone metastasis in both datasets (F_1_-score = 0.900 in the validation set and 0.847 in the additional-test set) (Table 2). Predicting extra-thoracic lymph nodes showed the lowest accuracy in the validation set (0.79) and the additional-test set (0.76) and predicting liver metastasis showed the highest accuracy in the validation set (0.97) and the additional-test set (0.97).

## Discussion

In this study, we developed a deep-learning model using the pseudo-label technique for extracting the primary site of lung cancer and metastatic lymph nodes and organs. Our deep-learning model had micro-AUROC values of 0.946 and 0.950 for predicting the primary cancer locations and metastatic organs in the additional-test set, and a sensitivity of 0.827 and a specificity of 0.960 for metastatic lymph nodes in the validation set. Although there are some concerns of low accuracy for predicting the primary cancer sites of huge left and huge right lobes, the model prediction for classifying between left and right lobes showed a modest degree of accuracy (96.4%). This technique could be used when searching for patients with unique metastatic status within the huge data warehouse at the hospital. To our knowledge, our research is the first to focus on extracting multiple information from radiology reports by implementing a semi-supervised learning method and we believe that this end-to-end framework could be further applied to other domains as well.

By utilising large patient datasets stored in electronic health records, various retrospective study designs can be conducted, although most of the semantic variables are identified as natural language and thus require laborious annotation. For example, in lung cancer, identifying metastatic status acquired by manual chart review is crucial in order to estimate the prognosis and severity of the disease. With the development and application of deep learning in the medical field, an increasing number of studies are being published on extracting information using the natural language processing technique. Although most of the predictions are focused on identifying the presence of a specific disease in radiologic reports. [23] Moreover, various types of data classes need to be extracted from radiology reports, which contain an abundance of medical information on the disease status. However, to our knowledge, there is a scarce amount of studies that investigated multiple labels required for lung cancer staging. In this study, we showed that our deep-learning model can extract multiple information from a radiology report for staging lung cancer, which may lead to the facilitation of studies requiring information on the lung cancer stage.

In this context, our model achieved a modest overall performance in the prediction of metastasis, although there were several instances of poor performance. In annotating primary lung lesions, any huge lesions could not be denoted as lobal locations, but rather had to be written as ‘left stump’ or ‘left central.’ This type of variation in written style makes pseudo-labelling a difficult task, which leads to the low accuracy for predicting huge left and right sites. However, considering that lung cancer staging only uses the information on whether the lobe is left or right, the overall accuracy would be preserved when predicting left or right in thoracic cages. In terms of distant metastasis prediction, our model had a modest predictive performance of around AUROC 0.95 in each label except for liver metastasis. When we reviewed the mispredictions of liver metastasis, the incidence of liver metastasis was smaller than that in the training set and had a different writing style. This kind of result can occur especially when only a small subset of data has positive labels in the training set and another validation set has a different writing style with a small subset of positive data. However, most of the other labels have similar writing styles or a modest number of positive data, thus leading to a modest performance in both data sets. With respect to lymph node metastasis, more positive labels in the training set led to the higher performance of prediction in our model. When predicting contralateral N1 and N2, the performance of the model was lower than other labels (F_1_-score of around 0.5). When reviewing the mispredictions, we noticed some tendencies of probable metastasis in ipsilateral N1 and N2 nodes, which could have led to the low performance of the model in predicting contralateral N1 and N2. Thus, identifying each metastasis information can be achieved by our model, although our model’s TNM staging could be less accurate when the N3 node is positive. Further study is therefore necessary to improve performance.

There were two major hurdles during the model implementation. First, even though the quality of data accounts for a considerable part in machine learning research, the quantity and the quality were not sufficient. The number of labelled data was limited, and some of the clinician-annotated data had errors such as the omission of positive labels. Annotation consistency was high in some variables, but not in liver metastasis or contralateral N1 (Additional file 1: Table S1). Second, as the writing style differs among medical experts, regularising and revising each data was a time- and labour-intensive process. However, pseudo-labelling enables the model to learn various writing styles by considering the underlying characteristics within the feature space of the labelled annotated data, which eventually leads to a good performance in most of the labels. As aforementioned, unlike the original pseudo-labelling that used probability for each label, our pseudo-labelling focused on keywords that have a great impact on determining the labels. We believe that it could be an answer to the lack of labelled data within medical fields.

This study has some limitations. First, as the datasets were collected from a single tertiary hospital, our model had not been evaluated by other hospitals’ reports which might have different writing styles by various radiologists. However, our methods are not based on the previously labelled dataset, but on a pseudo-label of the labelling style. If this method is used for another dataset in future work, the model could be evaluated whether this method could be generalised. Second, the model does not extract all metastatic sites or specific sites of metastasis. As the distribution of metastatic sites is skewed, rare or specific sites cannot make clusters that are sufficiently large for model training. To overcome this issue, the model can adjust the cut-off value for rare outcomes to reduce the number of false-positive results that lead to the overestimation of the tumour burden or tumour stages. With this method, the model can stably estimate the tumour burden with PET-CT label data.

Third, as the tumour stage such as size and invasion of the nearby structures, was not described in the radiologic reports, the T stage could not be determined by our model. This will need to be validated in chest CT reports, which will have more detailed information.

## Conclusion

We developed a deep-learning model that might be useful to extract information on primary sites and metastatic lymph nodes and distant organs from PET-CT radiology reports. Our method could be used for predicting the stage and tumour burden of lung cancer and may thus facilitate studies using electronic health record datasets by alleviating laborious annotations by clinicians.

## Supplementary Information

**Additional file 1.** The supplementary file provided detailed method and supplementary figures for comprehension of our research.

## Data Availability

The data that support the findings of this study are available from the institutional review board of Asan Medical Center, while restrictions apply to the availability of these data that were used under licence for the current study and so are not publicly available. However, data are available from the corresponding author upon reasonable request and with the permission of the institutional review board of Asan Medical Center.

## Abbreviations

PET-CT: Positron emission tomography-computed tomography
AUROC: Area under the receiver operating curve
AUPRC: And area under the precision-recall curve
LSTM: Long short-term memory model
NER: Named Entity Recognition
