# Supplementary material for: Automated extraction of information of lung cancer staging from unstructured reports of PET-CT interpretation: natural language processing with deep-learning
Source: BMC Med Inform Decis Mak. 2022 Sep 1;22:229. doi: 10.1186/s12911-022-01975-7 (PMC9438247; doi:10.1186/s12911-022-01975-7)
Supplement: Supplementary file 1 — Additional file 1. The supplementary file provided detailed method and supplementary figures for comprehension of our research. [file 12911_2022_1975_MOESM1_ESM.docx]

Additional Methods

Title: Automated extraction of information of lung cancer staging from unstructured reports of PET-CT interpretation: Natural language processing with deep learning.

Tables

[Additional Methods 3](#_Toc110684085)

[*Basic Pre-processing* 3](#_Toc110684086)

[*Lung Cancer Spell Checker (LCSC)* 3](#_Toc110684087)

[*Sentence-level segmentation* 5](#_Toc110684088)

[*NER-based Keyword Extraction* 6](#_Toc110684089)

[*Keyword extraction for primary sites of lung cancer* 8](#_Toc110684090)

[*Hyperparameter setting of our proposed model* 9](#_Toc110684091)

[*Evaluation of lung cancer spell checker* 9](#_Toc110684092)

[Additional Tables 11](#_Toc110684093)

[Table S1. Annotator’s consistency by Cohen’s kappa value. 11](#_Toc110684094)

[Table S2. Tag list for Named Entity Recognition 12](#_Toc110684095)

[Table S3. Notations used for primary site extraction 13](#_Toc110684096)

[Table S4. Confusion matrix for lung cancer spell checker 14](#_Toc110684097)

[Table S5. Examples of LCSC misclassification 15](#_Toc110684098)

[Figures 17](#_Toc110684099)

[Figure S1. Example of the conclusion section of a PET-CT report 17](#_Toc110684100)

[Figure S2. Extraction of keywords related to primary sites 18](#_Toc110684101)

[Figure S3. Rule-based postprocessing of extracted metastatic nodes 19](#_Toc110684102)

# **Additional Methods**

In this section, we describe the detailed modelling methods and how the pre-processing of raw sentences is treated. To correct the typographical errors, we implemented an auto-correcting algorithm called the Lung Cancer Spell Checker (LCSC) for all the raw sentences. As each sentence describe different metastatic sites, we assumed that all the sentences would have an independent meaning. This is why we implemented the RCNN model based on each sentence. Each TNM stage has distinct words depending on nodal and distant metastases. We therefore focused on each metastatic site with NER tagging. The following section presents a detailed description of this process, which was too large to be included in the main manuscript.

## *Basic Pre-processing*

Individual radiology reports were initially entered as inputs. We then implemented Regular Expression (RegEx) [1] in order to exclude any unnecessary special symbols. However, unlike regular text data, medical texts contain some special symbols that are particularly meaningful such as F/U (follow-up) and R/O (rule out). In these examples, removing the slash symbol (/) would not be appropriate. Therefore, we generated the RegEx query with the help of clinical experts in order to reflect the characteristics of medical data.

## *Lung Cancer Spell Checker (LCSC)*

After the basic pre-processing step, each input data point went through the LCSC to check for typos. The LCSC is a spelling correction tool for lung cancer-related domains and was developed using the *Symspell* model and released as an open-source in 2018 [2]. Most correction algorithms generally use all four techniques of delete, transpose, replace, and insert to analyse errors and misspellings; in contrast, *Symspell* has achieved significant improvements in terms of temporal and spatial complexity by only using the symmetric deletion technique.

To use this algorithm correctly, a word dictionary consisting of words and the frequency of each word is essential. In this study, for the purpose of optimizing the correction tool for the lung cancer domain, several lung cancer-related journals were used to analyse the words.

The implementation of the LCSC was as follows. First, a total of 608 words that appeared more than 60 times within our data were added to the word dictionary. Next, all lung cancer-related publications in journals or conferences were included by searching PubMed and PubMed Central. The search queries used in the process are as follows:

1. (((((((‘Lung cancer (Amsterdam, Netherlands)’[Journal]) OR ‘Journal of thoracic oncology: official publication of the International Association for the Study of Lung Cancer’[Journal]) OR ‘Clinical lung cancer’[Journal]) OR ‘Lung Cancer (Auckland, N.Z.)’[Journal]) AND hasabstract[text] AND ‘last 5 years’[PDat])) AND ‘last 10 years’[PDat])
2. (((‘Translational Lung Cancer Research’[Journal]) OR (Lung Cancer: Targets[Journal] AND Therapy[Journal])) OR ‘Lung Cancer Management’[Journal]) OR ‘Lung Cancer International’[Journal]

Query 1 returned 1,242 publications from PubMed and Query 2 returned 1,076 papers from PubMed Central. In all, we included 2,318 publications after removing redundant results and derived a total of 9,369 words that were mentioned more than 40 times among those papers. Finally, we generated a word dictionary containing 9,401 words.

We then trained the correction tool using *SymspellPy*, which is the Python version of *Symspell* [2]. The trained model can be finetuned for use in other medical domains by simply modifying the composition of the dictionary, similar to the aforementioned searching process. The following sentences show the results of the LCSC:

| (1)-A | **Metaatatic** lymphadenopathy in right **uppper** lower p**ratracheal**, **subcarinla**, left **interlobra** area |
| --- | --- |
| (1)-B | Metastatic lymphadenopathy in right upper lower paratracheal subcarinal left interlobar area |
| (2)-A | Hypermetabolic **noduel** found in left thyroid gland |
| (2)-B | Hypermetabolic nodule found in left thyroid gland |
|  |  |
| (3)-A | Hypermetabolic nodule **inright thyroidgland** |
| (3)-B | Hypermetabolic nodule in right thyroid gland |

(1)-A and (2)-A are sentences containing typos (bold-faced words). (1)-B and (2)-B are results from the LCSC where the underlined words have been corrected. (3)-A and (3)-B show examples with incorrect spacing, which were satisfactorily amended by our correction tool.

## *Sentence-level segmentation*

After fixing the potential typos within the input data, we separated each radiology report into sets of sentences. Sentence-level analysis can be helpful not only in the training phase but also during the result verification phase. First of all, using individual radiology reports as a whole would make it difficult for the neural network model to learn its vector representation. Because of the vanishing gradient problem [3] which turns all gradients into near-zero values within the Recurrent Neural Network (RNN), information in the early parts of the radiology reports might have less impact on its representation in the vector space. Eventually, regardless of the complexity of the network, the vanishing gradient problem could lead to decreases in accuracy. However, the shorter length of individual sentences compared with whole reports allows for more efficient training and improved time complexity.

## *NER-based Keyword Extraction*

After sentence-level segmentation, further action is necessary to reduce input data noise. Keyword extraction was performed by inputting each sentence that had been selected from the previous section. While analysing some of the input data, we noticed that the classification of most categories had a strong relationship with specific words. For example, when extracting the primary site, words related to a location such as ‘Left/Right, Upper/Middle/Lower’ had important information. Regarding distant metastasis, the appearance of the word ‘Bone’ was most often associated with ‘bone metastasis’ (Table S2). As such, since the words within each sentence confer significant influence on deciding its label, we designed the tags for NER in a way that includes the main keywords that constitute each category. The list of tags and corresponding keywords are shown in Table S2. Tags from Lung to Metastasis are sequentially labelled from 2 to 7, while the ‘Else’ tag is labelled as 1.

The following indicates the NER representation of a sentence:

| (1) | New metastatic lymph nodes in left upper paraesophageal paraaortic right prevascular areas |
| --- | --- |
| (2) | [1, 7, 6, 6, 1, 4, 5, 6, 6, 4, 6, 1] |

(1) was transformed into (2) as a sequence of numbers using the numbers corresponding to each label. For the purpose of dealing with different lengths of input data, we applied zero-padding to all the sentences. Specifically, because the longest sentence contained a total of 54 words, we represented each input into a sequence of 60 words and applied post-zero-padding.

The FastText algorithm [4] was used for word embedding. Unlike the existing distributed representation methods such as Word2Vec [5] or GloVe [6], sub-words were considered while learning the representation, which makes it relatively robust to typos. As such, if the spellings of the two words are similar to each other, they are generally projected to the nearest position in the vector space. For this reason, we anticipated that FastText could be the most suitable embedding method for use with medical texts, which in many instances contain an abundance of derivatives and compound words.

Each word vector was embedded in 100 dimensions and the learning rate was designated as 0.05. $Maxn$ and $Minn$, which are hyperparameters pertaining to character N-grams, were given the values of 5 and 3, respectively. Based on the trained model, each sentence was embedded into vectors with the shape of (60,100), where 60 refers to the maximum number of words within a single sentence and 100 refers to the embedding dimension. This vector served as our input, while the sequence of numbers in the above example was used as the output for the training step.

The network used for NER was a bi-directional LSTM network [7]. In order to overcome the problem of vanishing gradients in the previous single-way LSTM, this model optimised the weights by updating forwards as well as backwards. This input learns the optimized weights through the LSTM network by implementing both forward and backward propagation. $h_{t-1}^{\to}$ signifies the weights for the forward propagation at the time step $t-1$ and concatenates it with $h_{t-1}^{\leftarrow}$, which is the weight for the backward propagation, through the operation $\sigma$ that finally returns the output $y_{t-1}$. In our study, $y_{t-1}$ was a vector with shape (1,7) that contains the probability related to NER tags for the word $x_{t-1}$. In this context, the $Softmax$ function was used to derive the output in the form of a probability distribution. After the model training stage, we derived tags from each word within every sentence. During this process, we preserved all words except those classified with the ‘Else’ tag and the remaining words were considered the keywords of each sentence.

## *Keyword extraction for primary sites of lung cancer*

Table S3 shows the notations used for the primary site extraction phase. Each radiology report was divided into a set of sentences $S_{i}$. If the $i$^th^ input contained a total number of $j$ sentences, $S$ would include sentences ranging from $S_{i1}$ to $S_{ij}$. Next, the segmented sentences underwent a keyword extraction phase that returned the set $K$ that contained the list of keywords from $K_{i1}$ to $K_{ij}$, which is the same number as the components of $S$. Among the components of $K$, we excluded those not containing keywords with the ‘Lung’ tag to obtain list of $S$. ($LS$), which are sentences containing the ‘Lung’ tag words. As words with the ‘Lung’ tag mainly contain important findings related to the primary site, we focused solely on using $LS$ to reduce unnecessary noise within the input data.

Figure S2 illustrates our algorithm with actual input data. Figure 2-A is the original input data and Figure 2-B represents $S_{i}$. Figure 2-C shows the list of keywords for each component of $S_{i}$ and the underlined words account for the ‘Lung’ tag keywords. In this example, we can conclude that $S_{i1}$, $S_{i2}$, $S_{i5}$ $\in$ $LS$ and accordingly use $K_{i1}$, $K_{i2}$, and $K_{i5}$ for the prediction of primary sites. Similar to the NER process, the selected keywords are transformed into 100-dimensional vectors.

## *Hyperparameter setting of our proposed model*

The 1D-convolution was used for the convolution layer, and 50 nodes along with a stride length of 4 were also used. The width of the pooling operation was set to 2, and we set the dropout rate to 0.6 in order to prevent overfitting. After passing through LSTM layers containing 50 and 20 nodes, we applied additional dropout using a rate of 0.6. Finally, a fully connected layer with seven nodes followed by the $Softmax$ activation function was used to derive the outputs, giving the probability of being classified into one of the ‘Primary cancer locations’.

## *Evaluation of lung cancer spell checker*

In order to evaluate the performance of the spelling correction tool, we first identified all the sentences containing typos. Next, if a sentence contained typographical errors, it needed to be appropriately corrected, meaning that the precision of the model should be considered. For this reason, we first conducted experiments by focusing on identifying the existence of any typographical errors.

To begin with, the LCSC was applied to all the radiology reports. We selected 50 sentences containing typographical errors and 50 sentences without typographical errors, and this method enabled us to distinguish the sentences that would contain typographical errors. (Table S4). Table S5 shows examples of the errors in which correct words were replaced with incorrect words. The performance of the model was improved with the implementation of the LCSC and previous methods (Table S6).

Additional Tables

## **Table S1. Annotator’s consistency by Cohen’s kappa value.**

|  |  | Cohen’s kappa value |
| --- | --- | --- |
| N1 | Hilar | 0.83 |
|  | Interlobar | 0.84 |
|  | (Peri) Bronchial | 0.8 |
|  | Lobar | 0.44 |
| N2 | Upper paratracheal | 0.62 |
|  | Prevascular, retrotracheal | 0.62 |
|  | Lower paratracheal | 0.7 |
|  | Subaortic | 0.37 |
|  | Para-aortic | 0.61 |
|  | Subcarinal | 0.75 |
|  | Para-oesophageal | 0.71 |
| N3 | Contralateral N1 | 0.58 |
|  | Contralateral N2 | 0.74 |
|  | Supraclavicular | 0.86 |
| Metastasis | |  |
| Intrathoracic metastasis | Malignant pleural effusion | 0.79 |
|  | Malignant pericardial effusion | 0.66 |
|  | Pleural nodule | 0.79 |
|  | Contralateral lung | 0.74 |
|  | Ipsilateral lung | 0.64 |
|  | Synchronous lung cancer | 0.66 |
| Extra-thoracic metastasis | Lymphangitic meta | 0.84 |
|  | Bone (including rib and sternum) | 0.82 |
|  | Extra-thoracic lymph node | 0.8 |
|  | Brain | 0.91 |
|  | Adrenal | 0.83 |
|  | Liver | 0.58 |

## **Table S2. Tag list for Named Entity Recognition**

| **Tag** | **Implication** | **Examples** |
| --- | --- | --- |
| Lung | Words related to overall lung | Primary, Lesion, Lobe |
| Uncertainty^¶^ | Hedge/Weasel words | Small, Tiny, No, Without |
| Location 1 | Words related to Ipsilateral/Contralateral | Left, Right, Both |
| Location 2 | Words related to the position within the lobe | Upper, Mid, Lower |
| Lymph | Words related to lymph node metastasis  (N staging) | Lymph, Hilar, Interlobar |
| Metastasis | Words related to metastasis in other organs  (M staging) | Hypermetabolic, Bone |
| Else | Not included in any of the categories above | In, Biopsy, At, Size |

^¶^ When the lesion is obscure for malignant, the lesion is also annotated as an ‘Uncertainty’ lesion.

## **Table S3. Notations used for primary site extraction**

| $S_{ij}$ | $j$^th^ sentence in the $i$^th^ input data |
| --- | --- |
| $K_{ij}$ | Keyword lists for $S_{ij}$ |
| $LS$ | List of $S_{ij}$ where $K_{ij}$ contains words with the ‘Lung’ tag |

## **Table S4. Confusion matrix for lung cancer spell checker**

|  | **Modified** | **Not Modified** |
| --- | --- | --- |
| **With typographical errors** | 49 | 1* |
| **Without typographical errors** | 3* | 47 |

*One typo was not corrected and three corrected words were wrongly corrected by the LCSC.

## **Table S5. Examples of LCSC misclassification**

| **Misclassification Type** | **Original Sentence** | **Modified Sentence** |
| --- | --- | --- |
| Table 7-A | rec gastrosccopy if needed | rec gastric copy if needed |
| Table 7-B | rec t-spine mri | rec to spine mri |
| Table 7-B | pathologic compression fracture of t6 body with extension to spinal canal | pathologic compression fracture  or t6 body with extension to  spinal canal |
| Table 7-B | max suv=4.3 | max suv l3 |

**Table S6. Primary site classification accuracy with stepwise pre-processing**

| **Sentence Segmentation** | **LCSC (Spell Checker)** | **FastText** | **Keyword Extraction** | **Accuracy*** |
| --- | --- | --- | --- | --- |
| X | X | X | X | 0.5032 |
| O | X | X | X | 0.6228 |
| O | O | X | X | 0.7016 |
| O | O | O | X | 0.8577 |
| O | O | O | O | 0.9201 |

* Refers to the accuracy in predicting whether the primary cancer location originated from the left or right lobe.

# **Figures**

## **Figure S1. Example of the conclusion section of a PET-CT report**

**
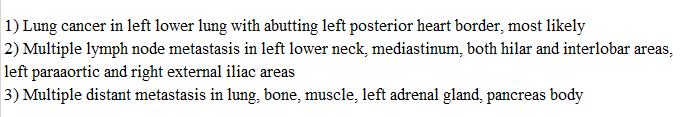
**

## **Figure S2. Extraction of keywords related to primary sites**


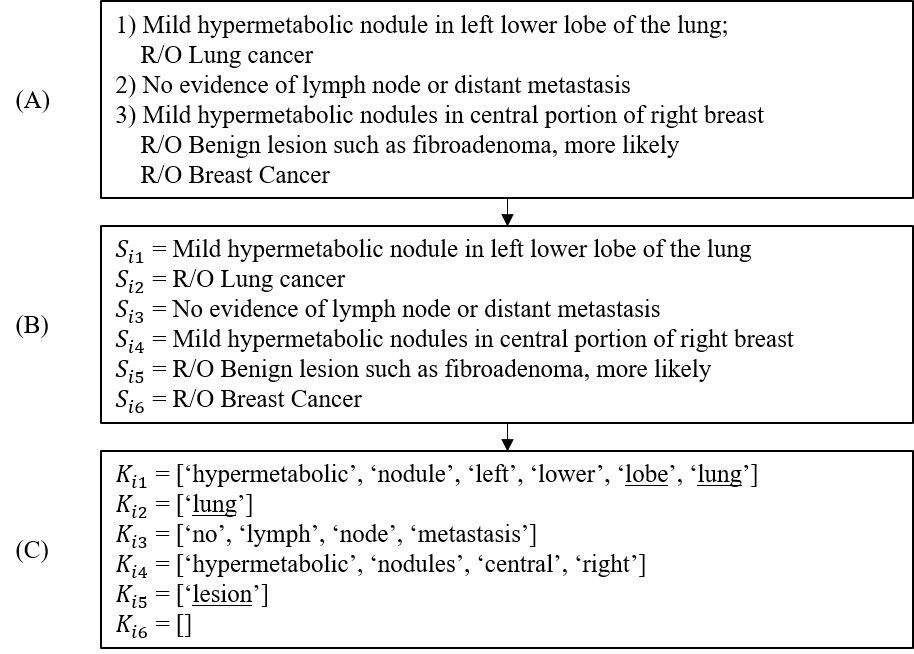


## **Figure S3. Rule-based postprocessing of extracted metastatic nodes**


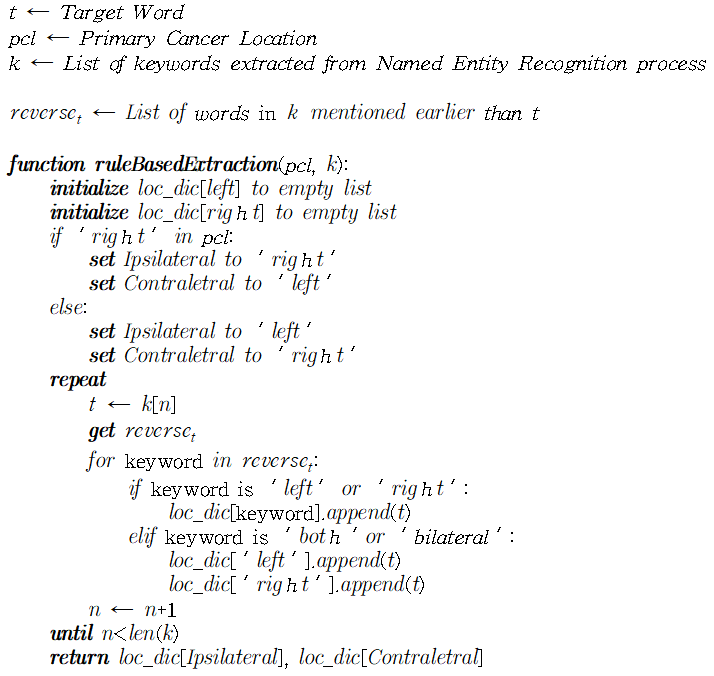


As the lymph node stage of lung cancer is determined by the primary sites of lung cancer, the location of each metastatic node was processed according to the flow.

**References**

1. Thompson K. Regular expression search algorithm. Comm ACM. 1968;11:419–22.

2. Garbe W. wolfgarbe/SymSpell: SymSpell: 1 million times faster spelling correction & fuzzy search through Symmetric Delete spelling correction algorithm. 2018. https://github.com/wolfgarbe/symspell. Accessed 28 Feb 2021.

3. Hochreiter S. The vanishing gradient problem during learning recurrent neural nets and problem solutions. Int J Uncertainty, Fuzziness Knowlege-Based Syst. 1998;6:107–16.

4. Bojanowski P, Grave E, Joulin A, Mikolov T. Enriching Word Vectors with Subword Information. Trans Assoc Comput Linguist. 2017;5:135–46.

5. Mikolov T, Sutuskever I, Chen K, Corrado G, Dean J. Distributed Representations of Words and Phrases and their Compositionality. Adv Neural Inf Process Syst. 2013;:1389–99.

6. Jeffrey P, Socher R, Manning CD. GloVe: Global Vectors for Word Representation Jeffrey. In: Conference on Empirical Methods in Natural Language Processing (EMNLP) 2014. 2014. p. 1532–43.

7. Schuster M, Paliwal KK. Bidirectional recurrent neural networks. IEEE Trans Signal Process. 1997;45:2673–81.
